# Imaging of Mouse Brain Fixated in Ethanol in Micro-CT

**DOI:** 10.1155/2019/2054262

**Published:** 2019-07-14

**Authors:** Jana Mrzílková, Matěj Patzelt, Pasquale Gallina, Zdeněk Wurst, Martin Šeremeta, Jan Dudák, František Krejčí, Jan Žemlička, Vladimír Musil, Jakub Karch, Jozef Rosina, Petr Zach

**Affiliations:** ^1^Specialized Laboratory of Experimental Imaging Third Faculty of Medicine, Charles University, Institute of Experimental and Applied Physics and Faculty of Biomedical Engineering, Czech Technical University in Prague, Prague, Czech Republic; ^2^Department of Anatomy, Third Faculty of Medicine, Charles University, Prague, Czech Republic; ^3^Department of Surgery and Translational Medicine, Neurosurgery Unit, Florence School of Neurosurgery, University of Florence, Florence, Italy; ^4^Institute of Experimental and Applied Physics, Czech Technical University, Prague, Czech Republic; ^5^Czech Technical University in Prague, Faculty of Biomedical Engineering, Kladno, Czech Republic; ^6^Centre of Scientific Information, Third Faculty of Medicine, Charles University, Prague, Czech Republic; ^7^Department of Medical Biophysics and Informatics, Third Faculty of Medicine, Charles University, Prague, Czech Republic

## Abstract

Micro-CT imaging is a well-established morphological method for the visualization of animal models. We used ethanol fixation of the mouse brains to perform high-resolution micro-CT scans showing in great details brain grey and white matters. It was possible to identify more than 50 neuroanatomical structures on the 5 selected coronal sections. Among white matter structures, we identified fornix, medial lemniscus, crossed tectospinal pathway, mammillothalamic tract, and the sensory root of the trigeminal ganglion. Among grey matter structures, we identified basal nuclei, habenular complex, thalamic nuclei, amygdala, subparts of hippocampal formation, superior colliculi, Edinger–Westphal nucleus, and others. We suggest that micro-CT of the mouse brain could be used for neurohistological lesions evaluation as an alternative to classical neurohistology because it does not destroy brain tissue.

## 1. Introduction

Microcomputed tomography (micro-CT) scanning provides nondestructive imaging of tissues and has potential to produce 3D images. Highly mineralized structures, such as bones and teeth, give very good contrast in micro-CT [1]. On the other hand imaging of soft tissues such as nerve, muscle, adipose tissue, or ligaments is very problematic [2]. Interestingly, alcohol fixation works well with the neuronal tissue and specifically with the brain but together with the iodine and phosphotungstic acid [3]. Brain tissue has several distinguishing characteristics compared to other soft tissues. It is composed of white matter that contains relatively high amount of phospholipid molecules forming myelin sheaths around axons of neurons that behave on the micro-CT simply as fat (mostly visualized on micro-CT darker compared to grey matter) and from grey matter containing bodies of neurons (appear on micro-CT lighter compared to white matter bundles) that share basic common cellular characteristics (biophysical, biochemical, and biological) of other soft tissues. Micro-CT studies visualizing neuronal tissue usually focus on peripheral nerves and their lesions [4], overall brain atrophy [5], freeze-dried human acellular nerve allografting (hANA) [6], and brain tumor models in mice [7]. Generally there are more micro-CT studies on pathological neuronal tissues than on the healthy ones.

Behavioral studies on the mice model often require precise analysis of the brain area selected for the experiment. For example, in animal models of ischemia exact place of the neuronal lesion has to be verified and quantified [8]. Evaluation of exact lesion site needs different kinds of structural/histological atlases ([9, 10]; Allen Mouse Brain Atlas, 2014) based on various staining procedures (Nissl, parvalbumin, calbindin, etc.). Besides classical neurohistological lesion verification, combination of 7T Bruker MRI with magnetic particle analysis (MPI) of the brain tissue in real time becomes popular [11]. Micro-CT imaging of the brain could be considered as a new attempt to visualize neuronal tissue for the experimental purposes. Micro-CT imaging with phase contrast of the ethanol fixated rat brain was successfully described in [12], although only gross neuroanatomical structures were observed. Soaking the brains in nonionic iodinated contrast agent resulted in clear differences in signal between the grey matter, the white matter, and the ventricular spaces [13], but without possibility to distinguish neuroanatomical borders of individual brain nuclei or cortical regions. Diffusible iodine-based contrast-enhanced computed tomography (diceCT) in female mouse was suggested to be effective for gross differences in the overall brain shape in large numbers of samples [14]. Combined MRI-CT atlases of developing and adult mouse brains fixed with paraformaldehyde and subsequent PBS wash-out are unique for coregistration of brain areas but without detailed neuroanatomical structures delineation [15]. We tried to visualize and identify on micro-CT as much as possible neuroanatomical structures on coronal, sagittal, and horizontal sections of the healthy mouse brain.

## 2. Materials and Methods

### 2.1. Tissue Sample Origins

We evaluated 5 brains from C57BL/6 genetically modified male mice (weight 17-20 g) from the Institute of Experimental Imaging, First Faculty of Medicine, Charles University, Prague, Czech Republic. This mouse strain was selected because it is commonly used in neurosciences and other research fields [16]. Mice were euthanized by cervical dislocation and their brains were harvested for purpose of this study; this method did not affect mice brain distortion at all. Study was approved by Ethical Committee of the Third Faculty of Medicine, Charles University, Czech Republic.

### 2.2. Tissue Sample Processing

Brains from 5 mice were carefully extracted from the skulls by the following steps. Cervical spinal cord and brain stem were released by small tongs as disruption of cervical vertebras. Then temporal bones and vestibulocochlear, oculomotor, optic, and olfactory nerves were dissected. After extraction of the brain from skull, any remnants of bone fragments on the brain surface were carefully checked and cleaned before scanning. The brain samples were put into Eppendorf tubes with ethanol-soaked gauze at the bottom for the purpose of the scan. The conical shape of Eppendorf tubes very efficiently supports the samples and avoids undesirable movements. The wet gauze maintains a saturated gaseous atmosphere preventing further drying out and shrinkage of sample. After the extraction, brains were fixated subsequently in 25%, 50%, 75%, and 97% ethanol for 12 hours. This type of ethanol fixation is also known as graded dehydration series of ethanol (GEHC) and has been documented as promising in undistorted soft tissue fixation [17]. Micro-CT scanning was performed after 168 hours of fixation.

### 2.3. Tissue Sample Scanning

Brains were left prior to scanning on the gauze for 40 minutes in air temperature 23°C. This allowed vaporization of redundant ethanol from the whole brain, including the ventricles and other cavities. After the period of drying, brains were positioned in the special plastic holder with an ethanol reservoir, which made an atmosphere of gas, which prevented structural changes of the brains during scanning [18, 19]. Two different scanning techniques were performed. First, just X-ray radiography was performed followed by a microtomography and final 3D reconstruction [18, 19]. The data were reconstructed into the final 3D dataset using Volex reconstruction software and visualized using program CTVox in standard PC [20]. On the sagittal projection some processing artifacts are often seen: flattening of the whole brain craniocaudally, artificial space between the hippocampal formation and thalamus, fimbria fornicis separated from the white matter nearby stria terminalis and ventriculus lateralis, and cerebellar fissure behind inferior colliculi.

### 2.4. Micro-CT Apparatus

The used micro-CT set-up was described in detail in our previous publications [18, 19, 21]. Briefly, apparatus consisted of two different custom-built micro-CT systems; routine detection system was equipped with a Kevex™ PXS-11 X-ray tube and Timepix detector in Quad configuration (four read-out chips with a common silicon sensor providing sensitive area 28 × 28 mm, 512 × 512 pixels, 55 *μ*m pixel pitch). The highest achievable spatial resolution was approximately 28 *μ*m. Presented 2D microradiographic images were acquired with the introduced setup. The other high-resolution system was equipped with a large area photon counting detector WidePIX_10×5_. WidePIX is a recently introduced technology for tiling of large area PCD arrays from individual Timepix chips [22]. Specifically, detector WidePIX_10x5_ is composed of 50 Timepix tiles and offers approximately 140 x 70 mm field view (2560 × 1280 pixels). High quality microfocus X-ray tube Hamamatsu L8601-01 enables spatial resolution down to 5 micrometers [23].

### 2.5. Scan Parameters

The high-resolution setup was used for the presented micro-CT scans. The data were acquired with an emphasis on high CNR. The acquisition time was adjusted in order to reach at least 10^5^ or 10^4^ detected photons per pixel in the background of the object in microradiography or a micro-CT projection, respectively. CT reconstructions were done by Volex reconstruction engine (courtesy of Fraunhofer IIS and Technology, Germany).

The detector as well as the whole CT scan was controlled using Pixelman software [24]. The CT scan was carried out with 4.4 *μ*m EPS. The total number of 848 projections was acquired with 0.38 degree angle step. The acquisition time was 3.6 seconds per projection. The tube voltage was set to 60 kVp and it was operated with 6 W of output power. The projections were processed using a dedicated beam-hardening correction [25] and the slight image distortions coming from the tiled detector construction were corrected [26]. The CT reconstruction was carried out using Volex reconstruction engine.

### 2.6. Gray and White Matter Labelling

For the frontal and sagittal sections Allen Mouse Atlas was used as reference [27]. For the horizontal sections C57BL/6J Atlas was used as reference (The Mouse Brain Library, http://www.mbl.org). Anatomical structures of digitalized brain sections were labeled in the environment of freeware program Xnview (https://www.xnview.com/en/). All depicted pictures of the labelled mouse brain are from one specimen only so that structures correspond between exactly three planes (horizontal, sagittal, and coronal), because of tiny morphological differences between various mouse brains.

## 3. Results

**Figure 1:**
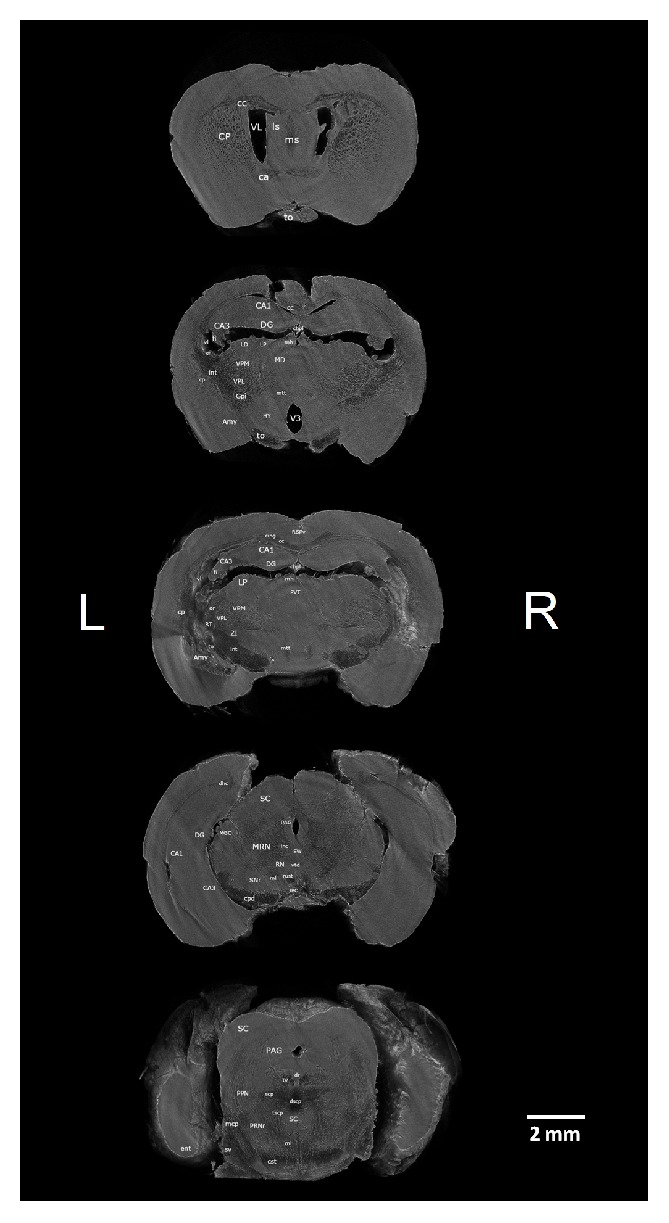
Micro-CT slices (program CTVox) of the 5 coronal sections of the mouse brain with labeled grey and white matter structures. Brain sections from top down are taken 1x at the level of anterior commissure, 2x at the dorsal hippocampus, 1x at the ventral hippocampus, and 1x at the brain stem (superior colliculi). Section orientation: L – left, R – right.

**Figure 2:**
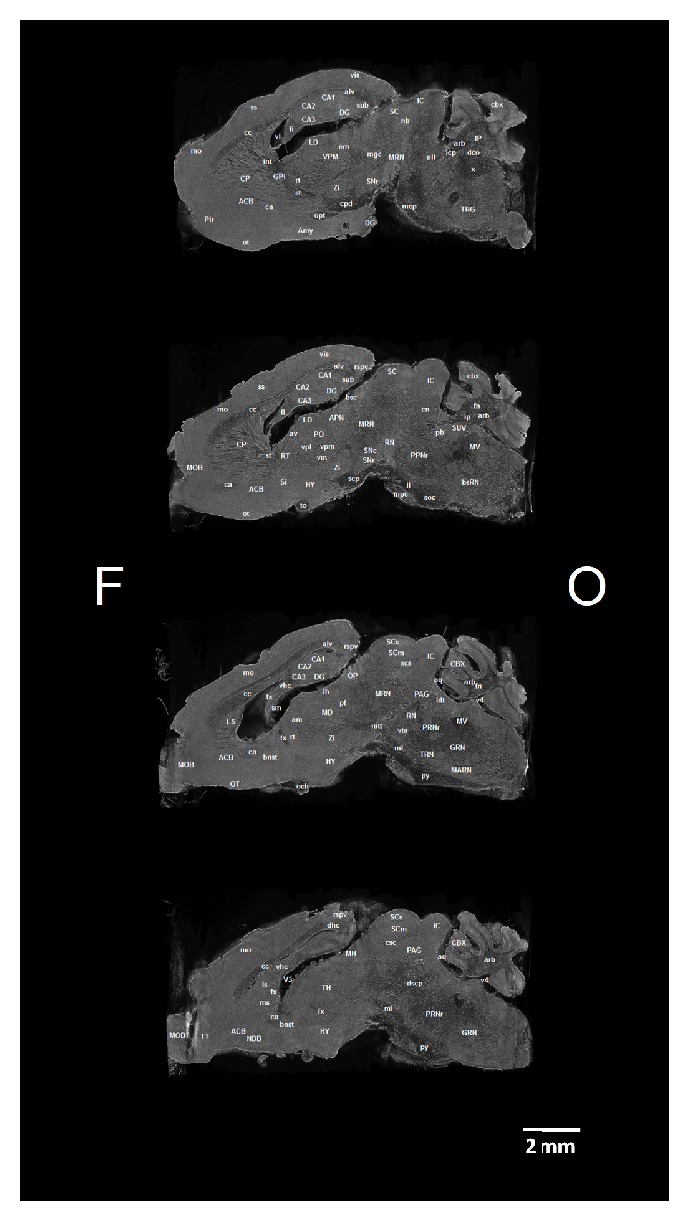
Micro-CT slices (program CTVox) of the 4 sagittal sections of the mouse brain with labeled grey and white matter structures. Brain sections from top down are taken 1x at the level of pallidum internum, 1x at the middle of caudatoputamen, 1x at the lateral septum, and 1x at the medial septum. Section orientation: F – frontal, O – occipital.

**Figure 3:**
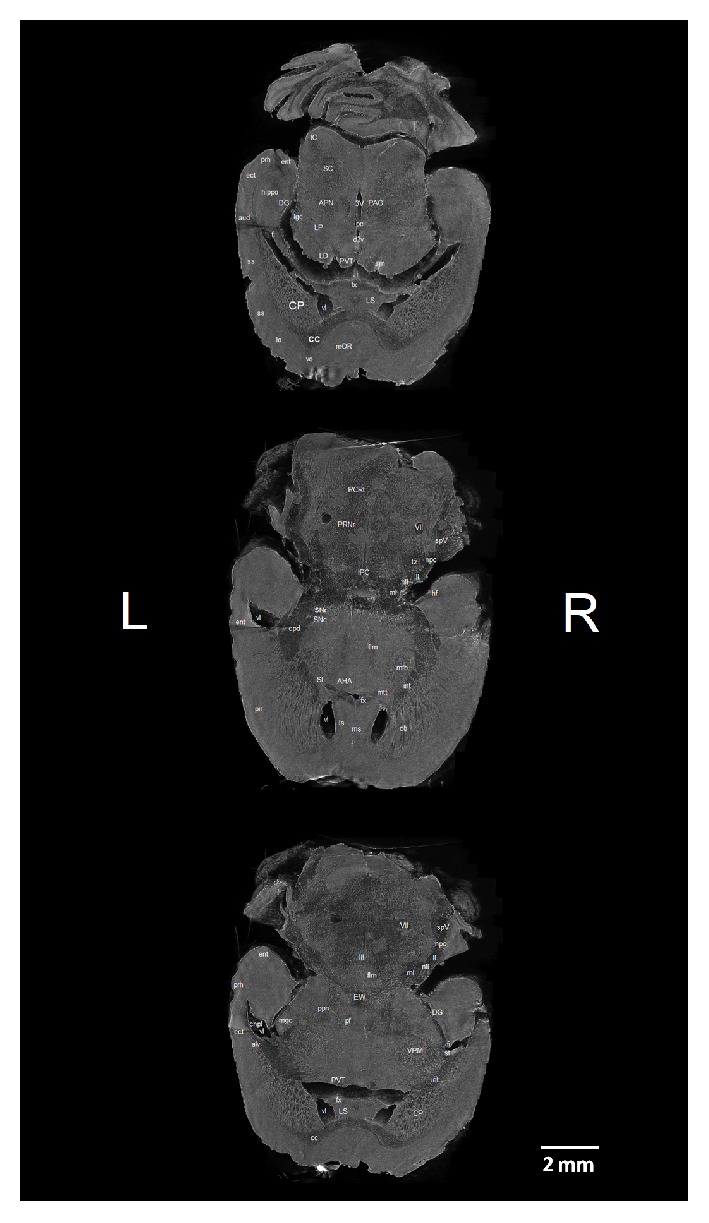
Micro-CT slices (program CTVox) of the 3 horizontal sections of the mouse brain with labeled grey and white matter structures. Brain sections from top down are taken 1x at the upper part of thalamic paraventricular nucleus, 1x at the anterior hypothalamic area, and 1x at the lower part of the thalamic paraventricular nucleus. Section orientation: L – left, R – right.

We identified 42 white matter and 53 grey matter brain structures (see Abbreviations) in five coronal (Figure 1), four sagittal (Figure 2), and three horizontal (Figure 3) brain sections of* ex vivo* healthy mouse brain using micro-CT. All structures were identified manually by two experienced neuroanatomists and registered in micro-CT scans using online Adult Mouse Brain Atlas [27] for coronal and sagittal sections and the online Mouse Brain Library (C57BL/6J Atlas) for horizontal sections.

### 3.1. Frontal Sections of the Mouse Brain

The positions of the five coronal sections of the mouse brain were selected because of their relevance to experimental neuropsychological studies in animal model. In frontodorsal order the sections were taken (a) in the frontal lobe at the level of the* anterior commissure*, (b) at the ventral part of the dorsal* dentate gyrus*,* dorsal hippocampus*, and the third ventricle, (c) at the dorsal part of the dorsal* dentate gyrus*,* dorsal hippocampus*, and the* paraventricular nucleus of the thalamus*, (d) at the level of the ventral* dentate gyrus*,* ventral hippocampus*, and* midbrain reticular nucleus*, and (e) at the level of the brain stem with* superior colliculi* and* dorsal raphe nucleus*. We were unable to identify hyperintensity in the brain stem between trigeminal nuclear complex, lateral lemniscal nuclei, and medial cerebellar peduncle. Another poorly visible area is located below superior and inferior colliculi, towards thalamic nuclei. Similarly, resolution of the bed nucleus striae terminalis and substantia innominata is poor. Opposite, there is good resolution for both zona incerta and reticular part of the substantia nigra. Although there is relatively big trigeminal nuclear complex that is easy to identify, separate subnuclei of the complex are hard to differentiate. The caudatoputamen is a very well visible structure and inside is rich system of the hypointensities that could be either white matter of the internal capsule or the Virchow-Robin spaces formed by capillary bed stream of thalamostriatal artery. Hypothalamic subparts are more difficult to discern compared to thalamic subnuclear groups. Stria medullaris thalami is normally found on the superior part of the thalamus; however here it is detached from it and bound to dorsal hippocampal commissure on the caudal surface of the fornix. While most of the thalamic nuclei are hyperdense, most midbrain structures (reticular nucleus, periaqueductal gray, etc.) are hypodense.

### 3.2. Sagittal Sections of the Mouse Brain

Within hippocampal formation dentate gyrus, CA1, Ca2, and CA3 subfields and subiculum were identified. Sagittal projection offers better visibility over frontal projection. Above cerebellar peduncle zona incerta, substantia nigra, and ventrally stria terminalis were visible. Within brain stem trigeminal nucleus, dorsal vagal nucleus, and nucleus interpeduncularis were visible. On the other hand, we have not seen well borders of the amygdaloid complex; it had the same gray color as nearby structures (olfactory tubercle or nucleus accumbens).

### 3.3. Horizontal Sections of the Mouse Brain

We identified in the ventral part of the sections clearly visible medial and lateral septum. Callosal body in contrast to online atlas was very well visible in all three sections and in front of it was well preserved medial frontal and/or orbital cortex. Also frontal part of the lateral ventricles clearly separated caudatoputamen from septal nuclei and internal capsule. Penetrations of the internal capsule into caudatoputamen are, especially, well visible. Dorsally to septal area structures of the thalamus (paraventricular nucleus and laterodorsal complex of nuclei) were located, and also stria medullaris thalami and lateral geniculate complex. On the other hand, detailed inner structure of the hippocampal formation (ventral part) was not very well visible as in the frontal sections. Besides reticular, pontine, or parvocellular nuclei, other brain stem nuclei were not visible compared to the frontal sections.

## 4. Discussion

Micro-CT imaging in mouse is often limited to the whole body scans, including skeleton, organs and blood vessels [28, 29], and brain blood supply changes in various experimental pathological conditions [30, 31], or to the brain tumors (for example glioblastoma) [32]. Scanning of the mouse brain gives better results when it is extracted from the skull. The reason is that the skull induces beam-hardening artifacts to adjacent soft tissue [15]. Our work provides comparable results as reported by [3]; nevertheless, in our case, any high-Z contrast agent was needed.

High-resolution MRI three-dimensional atlas of the mouse brain shows sixty-two structures at the resolution 32 *μ*m with the habenular nuclear complex being the smallest visible structure [33]. In comparison our micro-CT ethanol fixated brain scans showed more than fifty structures within only 5 representative coronal sections. It seems that pure GEHC ethanol brain fixation shows better differences between white and gray matters on micro-CT compared to MRI. We identified small white matter structures like cingulum bundle, medial lemniscus, crossed tectospinal pathway, and stria terminalis which is a better result compared to the MRI.

Frontal and sagittal sections atlases of the mouse brain [27] are easier to find in the literature compared to the horizontal ones (for example MRI atlas of C57BL/6J, DBA/2J, or A/J mouse from The Mouse Brain Library). Some MRI atlases are without grey and white brain matter labels, for example, 8-week-old 129S1/SvImJ male mice atlas [34].

We did not attempt yet to create the whole atlas of the mouse brain on the micro-CT. Our goal was to visualize clinically important brain structures like hippocampal formation and its subfields, thalamic nuclei, fornix, medial and lateral septal nuclei, and others. We suggest as the next step manual or semiautomatic reconstruction of the whole mouse brain micro-CT atlas. Histological staining (Nissl) and optical microscopy are mostly used for brain lesion evaluation in experimental studies (insertions of cannulas, electrolesions, chemical lesions, electrode positions, polymer substance delivery, ischemia after carotid arteries ligations, etc.). The disadvantage of these approaches is altered brain tissue that cannot be used afterwards for other staining (for example immunostaining) or at the cost of complicated protocols for sections storing and handling. With the micro-CT lesion verification, we can use intact brain tissue for further processing and thus replacing classical histological verification with virtual visual evaluation. Moreover, micro-CT lesion visualization can be enhanced by computer processing leading to volume rendering or providing virtual dissection of the brain in unorthodox planes unavailable in classical histology. Level of details in high-resolution micro-CT almost corresponds to the classical histology sections. Within destructive methodologies, it seems to be a choice for immunohistochemistry since the brains are processed only in ethanol.

## 5. Conclusion

We show that micro-CT could be used in neuroresearch alongside classical histology or magnetic resonance imaging. Besides higher price and lower resolution of the magnetic resonance imaging, it is not always available to all laboratories and micro-CT is easier to get access to. Even if one does not have micro-CT in the laboratory, it is possible to use fixation of the brain specimen and send it to micro-CT for analysis. This is not so simple for magnetic resonance imaging; we cannot use fixation or it would not be visualized properly. Fixation of the brain tissue should be done as soon as possible or the brain would decompose. Magnetic resonance imaging is better for living organisms while micro-CT for fixed brain tissue. Laboratories with micro-CT could offer services for others (sending fixated brain specimen) since acquisition time for micro-CT scanning is relatively short compared to magnetic resonance imaging. Immunohistochemistry or general staining histological protocols could then follow in a short time. The disadvantage of the micro-CT is still the relatively small Timepix detector area but with time we could expect an increase in its size. Ex vivo ethanol fixation of the brain tissue grants sufficient tissue contrast, but we trend to the situation where brain rotation could be highly contrasted even during in vivo scanning.

## Data Availability

Pictures of the mouse brain are available on request in our laboratory. The data used to support the findings of this study are available from the corresponding author upon request.

## Abbreviations

ACB: Nucleus accumbens
AHA: Anterior hypothalamic area
alv: Alveus
Amy: Amygdala
APN: Anterior pretectal nucleus
aq: Cerebral aqueduct
arb: Arbor vitae
aud: Auditory cortex
av: Anteroventral nucleus of the thalamus
bnst: Bed nucleus striae terminalis
bsc: Brachium of superior colliculus
bsRN: Brain stem reticular nuclei
ca: Anterior commissure
CA1: Field of CA1
CA3: Field of CA3
cb: Cell bridges of ventral striatum
cbx: Cerebellar cortex
cc: Corpus callosum
cing: Cingulum bundle
cn: Cuneiform nucleus
CP: Caudatoputamen
cpd: Cerebral peduncle
cst: Corticospinal tract
d3v: Dorsal third ventricle
dco: Dorsal cochlear nucleus
DG: Dentate gyrus
dhc: Dorsal hippocampal commissure
dr: Dorsal nucleus raphe
dscp: Superior cerebellar peduncle decussation
ect: Ectorhinal cortex
em: External medullary lamina of the thalamus
ent: Entorhinal area
EW: Edinger-Westphal nucleus
fi: Fimbria fornicis
flm: Medial longitudinal fascicle
fn: Fastigial nucleus
fx: Fornix
Gpi: Globus pallidus, internal segment
GRN: Gigantocellular reticular nucleus
hf: Hippocampal fimbria
hippo: Hippocampal formation
HY: Hypothalamus
chpl: Choroid plexus
IC: Inferior colliculus
icp: Inferior cerebellar peduncle
III: Oculomotor nucleus
inc: Interstitial nucleus of Cajal
int: Internal capsule
IP: Interposed nucleus of cerebellum
IPC: Interpeduncular nucleus
iv: Trochlear nucleus
LAV: Lateral vestibular nucleus
LD: Lateral dorsal nucleus of the thalamus
ldt: Laterodorsal tegmental nucleus
lgc: Lateral geniculate complex
lh: Lateral habenula
LHA: Lateral hypothalamic area
ll: Lateral lemniscus
lo: Lateral orbital cortex
LP: Lateral posterior nucleus of the thalamus
LPO: Lateral preoptic area
ls: Lateral septal nucleus
MARN: Magnocellular reticular nucleus
mcp: Middle cerebellar peduncle
MD: Mediodorsal nucleus of the thalamus
mfb: Medial forebrain bundle
MGC/mgc: Medial geniculate complex
mh: Medial habenula
ml: Medial lemniscus
mo: Somatomotor cortical areas
MOB: Main olfactory bulb
mOR: Medial orbital cortex
mpc: Medial cerebellar peduncle
MRN: Midbrain reticular nucleus
ms: Medial septal nucleus
mtt: Mammillothalamic tract
MV: Medial vestibular nucleus
MV: Medial vestibular nucleus
nb: Nucleus of the brachium of the inferior colliculus
NDB: Nucleus of the diagonal band
nll: Nucleus of the lateral lemniscus
not: Nucleus of the optic tract
och: Optic chiasma
OP: Olivary pretectal nucleus
or: Optic radiation
ot: Olfactory tubercle
PAG: Periaqueductal gray
pb: Parabrachial nucleus
pc: Posterior commissure
PCRt: Parvicellular reticular nucleus
pf: Parafascicular nucleus
Pir: Piriform cortical area
Po: Posterior thalamic complex
PPN/ppn: Pedunculopontine nucleus
ppt: Posterior pretectal nucleus
prh: Perirhinal cortex
PRNr: Pontine reticular nucleus
PVT: Paraventricular nucleus of the thalamus
py: Pyramid
RN: Red nucleus
RSPv/rspv: Retrosplenial area, ventral part
RT/rt: Reticular nucleus of the thalamus
rust: Rubrospinal tract
SC: Superior colliculus
SCm: Motor part of superior colliculus
scp: Superior cerebellar peduncles
SCs: Sensory part of superior colliculus
scs: Superior colliculus commissure
SI: Substantia innominata
sm: Stria medullaris thalami
SNc: Substantia nigra, compact part
SNr: Substantia nigra, reticular part
soc: Superior olivary complex
spV: Spinal tegmental tract
ss: Somatosensory cortical areas
st: Stria terminalis
stn: Subthalamic nucleus
sub: Subiculum
SUV: Superior vestibular nucleus
SV: Sensory root of the trigeminal nerve
TH: Thalamus
to: Tractus opticus
TRG: Trigeminal nuclei
TRN: Tegmental reticular nucleus
tscp: Crossed tectospinal pathway
TT: Taenia tecta
tz: Trapezoid body
V3: Third ventricle
v4: Fourth ventricle
vhc: Ventral hippocampal commissure
VII: Motor nucleus of the facial nerve
vIIIn: Vestibulocochlear nerve
vis: Visual cortical areas
VL/vl: Lateral ventricle
vm: Ventral medial nucleus of the thalamus
vo: Ventral orbital cortex
VPL: Ventral posterolateral nucleus of the thalamus
VPM/vpm: Ventral posteromedial nucleus of the thalamus
vta: Ventral tegmental area
vtd: Ventral tegmental decussation
x: Nucleus X
Zi/zi: Zona incerta.
